# The development of end stage renal disease in two patients with PMM2‐CDG

**DOI:** 10.1002/jmd2.12269

**Published:** 2022-01-10

**Authors:** Henna Tiwary, Leah E. Hecht, William J. Brucker, Gerard T. Berry, Nancy M. Rodig

**Affiliations:** ^1^ Boston Children's Hospital, Harvard Medical School Boston Massachusetts USA; ^2^ Hasbro Children's Hospital Providence Rhode Island USA

**Keywords:** CDG—chronic kidney disease, TLR—congenital disorder of glycosylation, PMM2—end stage renal disease, CKD—phosphomannosemutase‐2, ESRD—toll‐like receptor, UPC—urine protein‐to‐creatinine ratio

## Abstract

We report two patients with PMM2‐CDG who developed end stage renal disease (ESRD). Renal abnormalities of clinical significance have only been reported in about 6% of patients with PMM2‐CDG and have rarely been reported as the cause of death. Given the recurrent episodes of acute kidney injury associated with hospital admissions and the accelerated development of ESRD thereafter in our two patients, we recommend proactively involving Nephrology early in the care of these patients.

**Synopsis**

We report two patients with PMM2‐CDG who developed ESRD, and hope to bring attention to the potential importance of renal decline in PMM2‐CDG.

## 1 INTRODUCTION

The congenital disorders of glycosylation (CDG) are defects in glycan synthesis and in the proper attachment of glycans to proteins and lipids that results in multiple organ system involvement.^1^ The most prevalent congenital disorder of N‐glycosylation is PMM2‐CDG, which is a deficiency of the mannose pathway enzyme, phosphomannomutase‐2 (PMM2), due to mutations in the *PMM2* gene located on the 16p13.3 locus.^1^

Individuals with PMM2‐CDG have a host of clinical and laboratory abnormalities (refer to Table 1 and supplemental figures).^2^, ^3^ In this case report, we take a particular interest in the progression and degree of renal dysfunction present in patients with PMM2‐CDG. Glycoproteins that bind to the extracellular matrix and basement membrane play a significant role in developmental signaling and kidney organogenesis.^4^ Renal findings of clinical significance have only been reported in about 6% of this population.^3^ The most common congenital renal abnormality noted in this population is cystic kidney disease and the most common functional abnormality reported is mild proteinuria (mostly tubular in nature).^3^ Common findings in abnormal renal ultrasounds include renal cysts, increased echogenicity, enlarged kidneys, and altered corticomedullary differentiation. Of the most severe clinical renal manifestations seen is nephrotic syndrome, which has been reported in approximately less than 10 children in the existing literature.^3^ Data available for patients with proteinuria in the nephrotic range show that all patients presented within the first month of life and died within the subsequent 3 months, suggesting that renal abnormalities were likely congenital in nature. Other, less‐commonly reported renal abnormalities include acute kidney injury (AKI) and renal tubular dysfunction.^3^

In this report, we present two patients with PMM2‐CDG with normal renal function at birth and early infancy, who subsequently developed lethal end stage renal disease (ESRD). To the best of our knowledge, death associated with renal failure has only been reported once before in an infant with PMM2‐CDG and congenital nephrotic syndrome (Table 1).^5^

**TABLE 1:** The clinical phenotype of patients with PMM2‐CDG

	Existing literature	Case report	
		Patient 1	Patient 2
Common PMM2‐CDG, clinical findings			
Hypothyroidism	x	x	x
Pericardial effusion	x	x	x
Abnormal subcutaneous fat pattern	x	x	x
Global developmental delay	x	x	x
Renal findings			
Glomerular proteinuria (elevated urine microalbumin)	x	x	x
Tubulopathy (elevated urine beta‐2 microglobulin)	x	x	x
Nephrotic syndrome	x	x	
Acute kidney injury	x	x	x
Enlarged kidneys	x		
Renal cysts	x	x	x
Increased echogenicity	x	x	x
Abnormal corticomedullary differentiation	x	x	
Nephrocalcinosis			x
Calcification in renal tubules	x	*	*
Tubular dilatations	x	*	*
Mesangial matrix abnormalities	x	*	*
Hyaline casts in tubular lumen	x	*	*
End stage renal disease		x	x

*Note*: *, not assessed; x, present; not present.

## 2 CASE PRESENTATION

Patient 1 was a female with PMM2‐CDG who died at 4 years and 11 months of age, due to ESRD. Her renal manifestations included cystic renal disease and proteinuria. Her other medical issues at time of death included global developmental delay, hypotonia, abnormal liver function, protein‐losing enteropathy, hypoalbuminemia, pancytopenia, congenital hypothyroidism, hypoglycemia secondary to hyperinsulinism, retinal dystrophy, seizure disorder, bilateral moderate‐to‐severe hearing loss, coagulopathy, pericardial effusion, and eczema. *PMM2* gene sequencing confirmed the following compound heterozygote variants: p.Gly15Arg and p.Arg141His.

She was born at 37 weeks' gestation to a 27‐year‐old primigravida mother of European ancestry, who had an overall healthy pregnancy, and a father of African‐American ancestry. At 8 weeks of age, she was hospitalized due to failure to thrive and emesis. She was subsequently found to have elevated serum transaminases and hypoalbuminemia, resulting in a diagnosis of a congenital disorder of glycosylation. At 4 months, her renal ultrasound demonstrated increased echogenicity. Furthermore, she exhibited both tubular and glomerular proteinuria as evidenced by increased urine beta‐2 microglobulin and microalbumin, respectively.

At 7 months, she was hospitalized for a *Clostridioides difficile* infection and hypoglycemia. Her serum creatinine was normal at this time. A random urine protein to creatinine ratio (3.5, normal <0.2) and urine microalbumin (4100, normal <30 mg/g creatinine) were significantly elevated and serum albumin was decreased (2.4, normal 2.5–4.0 g/dl) with associated edema. She was provided supportive care with periodic albumin infusions to optimize volume status.

Between 12 and 14 months, her random urine protein to creatinine ratio, urine microalbumin, and urine beta 2‐microglobulin levels showed improvement.

At 24 months, she underwent extensive hospitalization for hypoglycemia due to hyperinsulinism and also experienced repeated episodes of pericardial effusion. Renal ultrasound at this time showed poor corticomedullary differentiation. At 26 months, she demonstrated decline in renal function with a serum creatinine of 0.7 mg/dl. Her glomerular proteinuria had mostly resolved at this time, however her tubular proteinuria was further exacerbated.

At 32 months, she was hospitalized for a febrile illness, presumed to be meningitis, and her serum creatinine increased to 1.1–1.2 mg/dl during this admission. At 36 months, she continued exhibiting tubular proteinuria, persistently creatinine, secondary hyperparathyroidism, and anemia, all believed to be related to her moderate‐to‐severe chronic kidney disease (CKD). A renal ultrasound indicated the development of numerous new bilateral renal cysts, consistent with a diagnosis of cystic dysplasia.

At 46 months, she developed pancytopenia and persistent deficiencies in Factor IX, Factor XI, antithrombin‐3, and protein C during a hospitalization for gastroenteritis, sinusitis, pulmonary nodular amyloidosis, and confirmed parvovirus infection. Consistent with an acute kidney injury, her serum creatinine level increased dramatically to 3.2 mg/dl with subsequent improvement. Despite a promising transient increase in all cell counts, she ultimately continued to exhibit trilinear pancytopenia.

In the final months of her life, she underwent multiple hospital admissions for various illnesses and gastrointestinal bleeding. Her serum creatinine continued to rise. Her progressive, multifactorial anemia and thrombocytopenia led to a greater susceptibility to GI bleeds that required repeated, regular transfusions of packed blood cells and platelets.

In her final days, treatment options of chronic dialysis and renal transplant were discussed with her parents given the severity of her CKD. After careful deliberation, her parents decided against pursuing renal replacement therapy and to focus on her quality of life. She was admitted to the hospital for pain control at 59 months for comfort care and passed away.

Patient 2 was a male with PMM2‐CDG who died at 11 years and 6 months of age due to ESRD. This patient was the subject of a previous report emphasizing perturbations in the vascular barrier that led to anasarca.^6^ Other medical issues at the time of death included diffuse hypotonia, seizure disorder, hypoglycemia, neuromuscular scoliosis, thrombocytopenia, coagulopathy, and cortical vision impairment. *PMM2* gene sequencing confirmed the following compound heterozygote variants: p. Gly42Arg and p. Pro113Leu.

He was born at full term to a 36‐year‐old primigravida mother of European ancestry, who had an overall healthy pregnancy, and a father also of European ancestry. During his first year of life, he presented low levels of protein C, protein S, anti‐thrombin, factor V, factor VII, factor IX, and factor XI—putting him at increased risk for clotting and bleeding. Furthermore, he presented an ongoing history of easy bruising, purpura and petechiae, a prominent genitalia fat pad, occasional epistaxis, osteopenia, and abnormal endocrine function including thyroid binding globulin deficiency.

**FIGURE 1:**
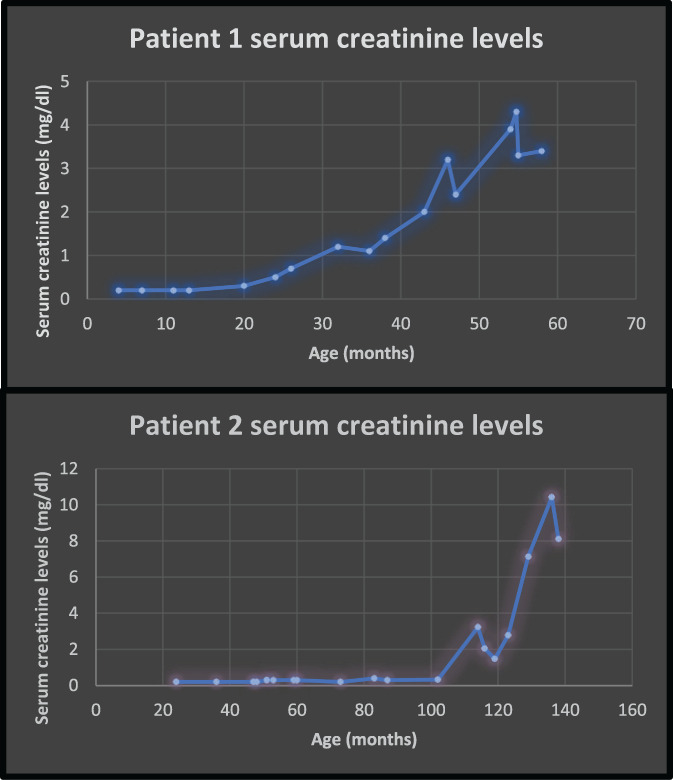
Serum creatinine levels of patients 1 and 2 over time

He showed evidence of increased renal echogenicity and renal cysts at 3 years of age. He also demonstrated mild persistent proteinuria, though his serum creatinine was maintained within the normal range until age 8 (refer to Figure 1 and supplemental tables for specific values). At age 4, prior renal echogenicity detected during his first year of life showed resolution, but existing renal cysts grew in size and he developed more cysts bilaterally. From ages 4–6, he continued to demonstrate both glomerular and tubular proteinuria.

At age 9, he required a 3‐month hospitalization for a life‐threatening and acute infection with pneumonia and disseminated intravascular coagulation. He developed multisystem organ failure with sepsis physiology associated with acute anuric renal injury and required 8 weeks of acute hemodialysis. During this time, he also required pericardiocentesis. His serum creatinine was approximately 0.3–0.4 mg/dl prior to the acute illness and rose to about 5 mg/dl at the time of initiation of continuous hemodialysis. At discharge and having been independent of dialysis for a 1 month, his serum creatine was approximately 2 mg/dl, indicating residual severe CKD. The advent of his hemodialysis treatment coincided with the onset of thrombocytopenia and bleeding complications—specifically mucosal and gastrointestinal bleeding. Given the severity of his CKD, he was expected to have progression to ESRD. At age 10 years, approximately 1 year after his severe acute illness, he initiated chronic hemodialysis for ESRD which he continued for the next year.

During his final months of life, he developed refractory thrombocytopenia and recurrent gastrointestinal bleeding. Given concerns for quality of life, the family elected to discontinue chronic hemodialysis. A final session of dialysis was scheduled by his family, and he passed away at 11 years and 6 months of age.

## 3 DISCUSSION

Renal anatomic and functional abnormalities are well described in patients with PMM2‐CDG. Patient 1 appeared to develop renal failure because of her underlying PMM2‐deficiency. She demonstrated an increase in renal echogenicity associated with normal renal function during early infancy but then progressed to ESRD over almost 5 years. Patient 2 also demonstrated early onset of increased renal echogenicity, renal cysts, and persistent proteinuria with maintained normal serum creatinine until age 8 years of age. In this patient, kidney failure was precipitated by sepsis physiology, and the contribution of underlying PMM2‐deficiency to progression of ESRD is uncertain.

It is well‐known that there is a high prevalence of altered immune function among patients with congenital disorders of glycosylation.^7^ Recurrent infections in childhood are commonly seen in patients with PMM2‐CDG, which tend to become less frequent in adolescence and adulthood,^8^ and recent findings on immunological dysfunction show that viral infections have been associated with 42% of stroke‐like episodes in children with PMM2‐CDG.^9^ When considering etiologies of renal injury and subsequent decline in renal function in our two patients, deficits in the glycan assembly of certain immunological factors may have increased the risk of recurrent illnesses and secondary acute kidney injury (AKI). Given that both patients 1 and 2 developed AKI while receiving treatment for gastroenteritis and pneumonia, respectively, we hypothesize that compromised immune function characteristic of PMM2‐CDG may have played a role in exacerbating the progression of kidney disease. Specifically, the pneumonia of patient 2 led to multisystem organ failure with sepsis physiology and ultimately resulted in anuric AKI. The complex mechanism of AKI associated with sepsis includes renal hypoperfusion, ischemic tubular epithelial cell injury, and acute tubular necrosis.^10^ For example, renal tubular epithelial cells express the Toll‐like receptors TLR2 and TLR4, which are responsible for initiating a cascade of signals that encourage the synthesis and release of proinflammatory molecules.^10^ Inflammatory dysregulation, such as that seen in sepsis, may be partially responsible for organ dysfunction and abnormalities seen in affected tissues.^10^ These mechanisms associated with AKI would undoubtedly exacerbate any underlying tubular perturbations, which are commonly seen in patients with PMM2‐CDG, and may worsen the long‐term trajectory of renal function.

Other possible explanations for the development of ESRD include preliminary data suggesting that patients with PMM2‐CDG demonstrate capillary leak, even when they do not have an infection or another acute illness.^6^ In addition to capillary leak, other factors such as insufficient mannose availability during embryonic development may lead to hypoglycosylation, abnormal tissue morphology, and subsequent multiple organ degradation.^3^ Preliminary studies on mice with PMM2‐CDG bring attention to the potential role of mannose supplementation in rescuing N‐linked glycoproteins during the prenatal stage, and such studies might be worth expanding upon in the case of humans with PMM2‐CDG who develop ESRD.

The etiology of renal failure in both patients still remains unknown. In the case of van der Knaap et al. an infant born with PMM2‐CDG and congenital nephrotic syndrome died at 2 months of age associated with a multi‐organ failure which included renal failure.^5^ This infant is quite different from our patient 1, and more like patient 2 in whom multiple organ systems were impaired. More studies need to be performed to identify the relevant etiology that may aid in establishing better treatment protocols to minimize or eliminate this complication. Based on the development of ESRD in our two pediatric patients with PMM2‐CDG, we recommend an emphasis on early involvement of nephrologists in the multidisciplinary care of PMM2‐CDG patients. Close follow‐up and evaluation of renal markers during and after episodes of illness can help to catch early signs of progressive renal decline. Optimal care may include supportive measures such as avoiding nephrotoxic exposures, when possible, in patients with PMM2‐CDG during episodes of acute infection to reduce the risk of AKI and progressive renal injury. This would be particularly prudent in those with a history of AKI or known CKD (i.e., persistent proteinuria, abnormal renal imaging, and/or abnormal renal function). Lastly, we hope to encourage future research into the additional genetic risk factors that may interact with CDG mutations and contribute to the accelerated development of system‐specific organ failure. We hope that this apparent novel presentation of ESRD in PMM2‐CDG will encourage investigation into the pathophysiology of N‐linked glycosylation disorders in kidney organogenesis, and ultimately lead to better awareness, treatment, and prevention of renal decline in patients with PMM2‐CDG.

## CONFLICT OF INTEREST

The authors declare that they have no conflicts of interest.

## AUTHOR CONTRIBUTIONS

Gerard T. Berry, Nancy Rodig, William Brucker, and Leah Hecht followed these patients and were involved in their care until the end of their lives. Henna Tiwary drafted this manuscript and Gerard T. Berry revised this manuscript, along with the help of Nancy Rodig, Leah Hecht, and William Brucker.

## ETHICS APPROVAL

An Ethics Approval Statement was not required for this report.

## ANIMAL RIGHTS

This article does not contain any studies with human or animal subjects performed by the any of the authors.

## PATIENT CONSENT

All procedures followed were in accordance with the ethical standards of the responsible committee on human experimentation (institutional and national) and with the Helsinki Declaration of 1975, as revised in 2000 (5). Informed consent was obtained from all patients and/or guardians for being included in the study. Additional informed consent was obtained from all patients for which identifying information is included in this article.

## Supporting information


**Data S1**: Supporting Information.

## Data Availability

The data that support the findings of this study are available from the corresponding author upon reasonable request.
