# Supplementary material for: The development of end stage renal disease in two patients with PMM2‐CDG
Source: JIMD Rep. 2022 Jan 10;63(2):131–6. doi: 10.1002/jmd2.12269 (PMC8898725; doi:10.1002/jmd2.12269)
Supplement: Supplementary file 1 — Data S1: Supporting Information. [file JMD2-63-131-s001.docx]

1. **Supplemental Figures**

See attached pages.

Figure 1S. Urine Microalbumin Levels of Patients 1 and 2 over time

Figure 2S. Urine Beta 2-Microglobulin Levels of Patients 1 and 2 over time

Figure 3S. eGFR values for Patients 1 and 2 over time


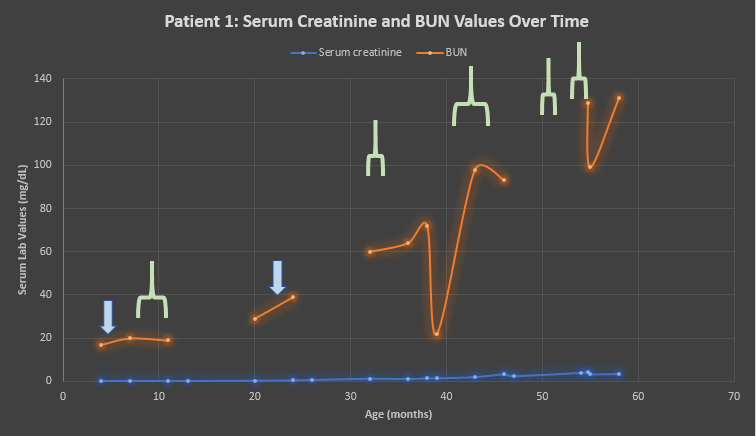


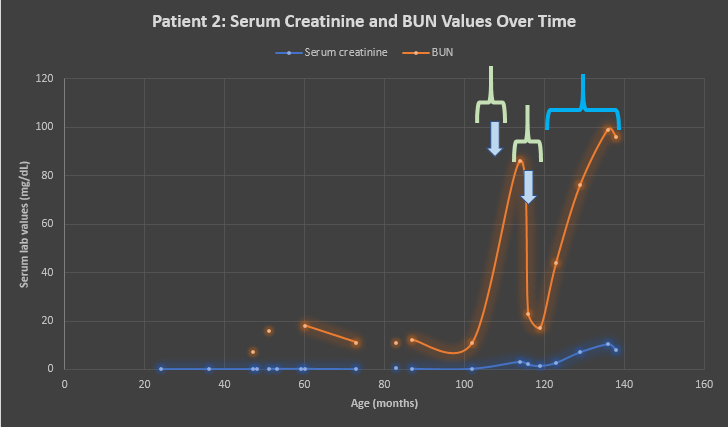


Figure 4S. BUN and serum creatinine values for Patients 1 and 2 over time

| Key | |
| --- | --- |
| bracket | FFP/other blood product transfusion + dialysis |
| bracket | FFP/other blood product transfusion |
| arrow | albumin infusion |

| Age (months) | Serum Creatinine (mg/dL) | BUN (mg/dL) | Urine microalbumin (mg/g creatinine) | Urine beta-2 microglobulin (mcg/g creatinine) | Urine protein-to-creatinine ratio | Serum Albumin (g/dL) |
| --- | --- | --- | --- | --- | --- | --- |
| 4 | 0.2 | x | 2854 | 1842 | 3.6 | 2.8 |
| 7 | 0.2 | 17 | 4100 | x | 3.5 | 2.4 |
| 11 | 0.2 | 20 | x | x | 1.25 | 3.3 |
| 13 | 0.2 | 19 | x | x | 0.57 | x |
| 14 | x | x | 110.5 | 1345 | 0.55 | 3.9 |
| 20 | 0.3 | 29 | 44 | x | 0.44 | 4 |
| 24 | 0.5 | 39 | 152.8 | 1046 | 0.75 | 3.9 |
| 26 | 0.7 | x | 21.7 | 2107 | 0.3 | x |
| 32 | 1.2 | 60 | x | x | x | 3.1 |
| 36 | 1.1 | 64 | x | x | x | x |
| 38 | 1.4 | 72 | x | x | x | x |
| 39 | 1.4 | 22 | x | x | x | x |
| 43 | 2 | 98 | x | x | x | x |
| 46 | 3.2 | 93 | x | x | x | x |
| 47 | 2.4 | x | x | x | x | x |
| 54 | 3.9 | x | x | x | x | x |
| 54.75 | 4.3 | 129 | x | x | x | x |
| 55 | 3.3 | 99 | x | x | x | x |
| 58 | 3.4 | 131 | x | x | x | x |

Table 1S. Patient 1 Lab History

| Age (months) | Serum Creatinine (mg/dL) | BUN (mg/dL) | Urine microalbumin (mg/g creatinine) | Urine beta-2 microglobulin (mcg/g creatinine) | Urine protein-to-creatinine ratio | Serum Albumin (g/dL) |
| --- | --- | --- | --- | --- | --- | --- |
| 24 | 0.2 | x | x | x | x | x |
| 36 | 0.2 | x | x | x | x | x |
| 47 | 0.2 | 7 | 466 | x | x | x |
| 48 | 0.2 | x | x | x | x | x |
| 50 | X | x | 569 | 459 | 1 | x |
| 51 | 0.3 | 16 | x | x | x | 4 |
| 53 | 0.3 | x | x | x | x | x |
| 59 | 0.3 | x | 398 | x | x | x |
| 60 | 0.3 | 18 | x | x | x | 4.2 |
| 73 | 0.2 | 11 | 120 | 899 | x | 4.4 |
| 78 | X | x | 94.2 | 1198 | 0.3 | x |
| 83 | 0.4 | 11 | x | x | x | 4 |
| 86 | X | x | 315 | 1832 | 0.59 | x |
| 87 | 0.3 | 12 | 92.1 | x | 0.3 | 4.1 |
| 102 | 0.3 | 11 | 55.5 | 860 | 0.29 | 4.3 |
| 114 | 3.2 | 86 | x | x | x | 3.1 |
| 116 | 2.1 | 23 | x | x | x | x |
| 119 | 1.5 | 17 | x | x | 2.63 | x |
| 123 | 2.8 | 44 | x | x | x | 3.6 |
| 129 | 7.1 | 76 | x | x | x | 3.2 |
| 136 | 10.4 | 99 | x | x | x | 4.9 |
| 138 | 8.1 | 96 | x | x | x | 5.1 |

Table 2S. Patient 2 Lab History

| The Biochemical Phenotype of Patients with PMM2-CDG | | | | |
| --- | --- | --- | --- | --- |
| Common PMM2-CDG  Laboratory Findings | **Existing Literature** | | **Case Report Patients** | |
|  | **Child** | **Adult/Adolescent** | **Patient 1** | **Patient 2** |
| Elevated transaminases (hepatic dysfunction) | x | x | x | x |
| Abnormal prothrombin time (coagulopathy) | x |  |  |  |
| Low serum concentrations of: factor IX, factor XI, antithrombin III, protein C, and/or protein S (coagulopathy) | x | x | x | x |
| Hypoglycemia (some due to hyperinsulinism) | x |  | x | x |
| Elevated TSH (hypothyroidism) | x |  | x | x |
| Normal or abnormal calcium, magnesium, and phosphate levels (osteopenia) | x |  | x | x |
| Elevated creatinine (proteinuria and aminoaciduria) | x |  | x | x |
| Varying levels of FSH, LH, and estradiol (gonadal dysfunction) |  | x |  |  |
| Low testosterone and sex-binding globulin (gonadal dysfunction) |  | x |  |  |

Table 3S. The Biochemical Phenotype of Patients with PMM2-CDG

| The Clinical Phenotype of Patients with PMM2-CDG | | | | | |
| --- | --- | --- | --- | --- | --- |
| Most Common PMM2-CDG Clinical Findings | **Existing Literature** | | | **Case Report** | |
|  | **Fetus** | **Child** | **Adult/Adolescent** | **Patient 1** | **Patient 2** |
| Nonimmune hydrops fetalis | x |  |  |  |  |
| Growth abnormalities |  | x |  | x | x |
| Global developmental delay |  | x |  | x | x |
| Hypothyroidism |  | x |  | x | x |
| Hypogonadism |  | x |  |  |  |
| Esotropia |  | x |  |  | x |
| Pericardial effusion |  | x |  | x | x |
| Abnormal subcutaneous fat pattern |  | x |  | x | x |
| Seizures |  | x | x | x | x |
| Stroke-like episodes |  | x | x |  |  |
| Recurrent infections |  | x |  | x | x |
| Vaccine non-responsiveness |  | x |  |  |  |
| Osteopenia |  | x |  | x | x |
| Scoliosis |  | x |  |  | x |
| Cerebellar hypoplasia/atrophy |  | x |  |  |  |
| Cerebellar dysfunction |  |  | x |  |  |
| Non-progressive cognitive impairment |  |  | x |  |  |
| Peripheral neuropathy |  |  | x |  |  |
| Atypical secondary sexual development |  |  | x |  |  |
| Retinal Dystrophy |  |  | x | x |  |
| Myopia |  |  | x | x |  |
| Joint contractures |  |  | x |  |  |
| Renal Findings | **Existing Literature** | | | **Patient 1** | **Patient 2** |
| Glomerular Proteinuria (elevated urine microalbumin) | x | | | x | x |
| Tubulopathy (elevated urine beta-2 microglobulin) | x | | | x | x |
| Nephrotic syndrome | x | | | x |  |
| Acute Kidney Injury | x | | | x | x |
| Enlarged kidneys | x | | |  |  |
| Renal cysts | x | | | x | x |
| Increased echogenicity | x | | | x | x |
| Abnormal corticomedullary differentiation |  | | | x |  |
| Nephrocalcinosis | x | | |  | x |
| Calcification in renal tubules | x | | | * | * |
| Tubular dilatations | x | | | * | * |
| Mesangial matrix abnormalities | x | | | * | * |
| Hyaline casts in tubular lumen | x | | | * | * |
| End Stage Renal Disease |  | | | x | x |

Table 4S. The Clinical Phenotype of Patients with PMM2-CDG (expanded)

| Key | |
| --- | --- |
| * | not assessed |
| x | present |
|  | not present |
